# Involvement of CXCL12/CXCR4 in the motility of human first-trimester endometrial epithelial cells through an autocrine mechanism by activating PI3K/AKT signaling

**DOI:** 10.1186/s12884-020-2788-3

**Published:** 2020-02-10

**Authors:** Jiayi Zheng, Danni Qu, Chen Wang, Ling Ding, Wenhui Zhou

**Affiliations:** Medical Center for Human Reproduction, Beijing Chaoyang Hospital, Capital Medical University, Beijing, 100020 People’s Republic of China

**Keywords:** Endometrial epithelial cell (EEC), Motility, CXCL12/CXCR4, Phosphorylation, Maternal-fetal interface

## Abstract

**Background:**

CXCL12(chemokine ligand 12, CXCL12) and its receptors CXCR4 are widely expressed in maternal-fetal interface and plays an adjust role in materno-fetal dialogue and immune tolerance during early pregnancy. This study aimed to evaluate the role and mechanism of self-derived CXCL12 in modulating the functions of human first-trimester endometrial epithelial cells (EECs) and to identify the potential protein kinase signaling pathways involved in the CXCL12/CXCR4’s effect on EECs.

**Methods:**

The expression of CXCL12 and CXCR4 in EECs was measured by using immunohistochemistry, immunofluorescence, real-time polymerase chain reaction and enzyme-linked immunosorbent assay. The effects of EEC-conditioned medium (EEC-CM) and recombinant human CXCL12 (rhCXCL12) on EEC migration and invasion in vitro were evaluated with migration and invasion assays. In-cell western blot analysis was used to examine the phosphorylation of protein kinase B (AKT), extracellular regulated protein kinases (ERKs) and phosphatidylinositol 3-kinase (PI3K) after CXCL12 treatment.

**Results:**

CXCL12 and CXCR4 were both expressed in human first-trimester EECs at the mRNA and protein level. Both EEC-CM and rhCXCL12 significantly increased the migration and invasion of EECs (*P* < 0.05), which could be blocked by neutralizing antibodies against CXCR4 (*P* < 0.05) or CXCL12 (P < 0.05), respectively. CXCL12 activated both PI3K/AKT and ERK1/2 signaling and CXCR4 neutralizing antibody effectively reduced CXCL12-induced phosphorylation of AKT and ERK1/2. LY294002, a PI3K-AKT inhibitor, was able to reverse the promotive effect of EEC-CM or rhCXCL12 on EEC migration and invasion.

**Conclusions:**

Human first-trimester EECs promoted their own migration and invasion through the autocrine mechanism with CXCL12/CXCR4 axis involvement by activating PI3K/AKT signaling. This study contributes to a better understanding of the epithelium function mediated by chemokine and chemokine receptor during normal pregnancy.

## Background

Successful embryo implantation and pregnancy maintenance is dependent on a receptive endometrium and close coordination between the endometrium and the conceptus [1–3]. Changes in endometrial receptivity and the aberrant expression of endometrial factors are associated with recurrent implantation failure and miscarriage [4–6]. It is now acknowledged that endometrial receptivity and function are the main determinants of a successful pregnancy [7–9]. However, the mechanisms underlying the modulation of endometrium function have not been fully elucidated.

Numbers of signaling molecules are expressed in the endometrium during early pregnancy. Among them, chemokines are unique proteins with the ability to control immune cell chemoattraction and participate in cell proliferation, migration as well as apoptosis. Chemokine ligand 12 (CXCL12) can modulate proliferation, invasion and survival of human trophoblasts, which is crucial for the establishment of a successful pregnancy [10–12]. CXCL12 and its receptor CXCR4 are involved in uterine natural killer cell recruitment, placentation, vessel remodeling, embryogenesis and cardiovascular and central nervous system organogenesis [10–16]. Dysregulation of CXCL12 is associated with pregnancy complications such as intrauterine growth restriction and preeclampsia [17, 18]. It is instructive to explore the function of CXCL12/CXCR4 in the establishment and functioning of a receptive endometrium.

As the first maternal-derived cells to interact with the fetus-derived trophoblast cells (TCs), endometrial epithelial cells (EECs) are an intrinsic component of a receptive endometrium and involved in many essential biological processes during pregnancy [19–24]. Although the importance of EECs at the materno-fetal interface is well acknowledged, the characteristics and functions of EECs remain largely unknown. During the process of trophoblast invasion, the gland and EECs must have their own unique functions. It has been suggested that an insufficient uterine glands function is correlated to some pregnancy-related diseases, including recurrent implantation failure, recurrent miscarriage, pre-eclampsia, fetal growth restriction [19–24].

Our previous studies supported the involvement of CXCL12/CXCR4 signaling in the coordination of fetal-derived TCs and decidual stromal cells (DSCs) during implantation and early pregnancy [25–28]. However, our previous researches mainly focused on the role of TCs-derived CXCL12 in the modulation of maternal-derived cells. Whether EECs secrete CXCL12 and the regulation of CXCL12/CXCR4 axis in EECs can be achieved in an autocrine manner is still an open question. CXCL12/CXCR4 can promote cells proliferation, migration and invasion. Motile ability is the basic function of cells during invasion. Thus, we suppose that the endometrium might be dynamically motile and make positive adjustments rather than merely passively waiting for embryo implantation during TCs invasion.

In the present study, we used CXCR4 as control and measured CXCL12 expression in human first-trimester EECs and then explored the effects of self-derived CXCL12 on EEC invasion and migration; furthermore, we also explored the potential protein kinase signaling pathways involved in the effect of CXCL12/CXCR4. The purpose of this study is to improve our understanding on the underlying molecular mechanisms involved in EECs functions and the establishment of the maternal-fetal interface during implantation and pregnancy.

## Methods

### Tissue collection

Human decidual tissue samples were collected from patients (aged between 20 to 40 years) who underwent elective surgical abortion during first-trimester pregnancy (gestational age 8–10 weeks) due to unintended pregnancy for nonmedical reasons. Samples were collected and immediately put into ice-cold DMEM (high D-glucose) (Dulbecco’s modified Eagle medium, Gibco, Grand Island, NY, USA) and then transported to the laboratory within 1 hour (h) of collection for EEC isolation or paraffin embedding [25, 26].

### Immunohistochemistry

The decidual tissues was fixed with 4% paraformaldehyde for 4 h and embedded with paraffin. Then paraffin-embedded decidual tissues was serially sectioned (thickness 5 μm). After deparaffinization and rehydration with xylene and gradient alcohol, antigenic recovery was performed by microwave heating in 0.01 M sodium citrate buffer (pH 6.0). H2O2 (0.3%) in phosphate-buffered saline (PBS) was used to block endogenous peroxidase activity in the sections. Then the sections were treated with protein blocking solution containing 5% bovine serum albumin to block non-specific binding and then incubated with mouse anti-human CXCL12 (10 μg/ml, MAB 350, R&D Systems, Abingdon, UK) or CXCR4 (25 μg/ml, MAB172, R&D Systems, Abingdon, UK) overnight at 4 °C. After three washes with PBS, the sections were overlaid with peroxidase-conjugated goat anti-mouse IgG (SP-9002; SZGB-BIO, Beijing, China), followed by 3,3-diaminobenzidine tetrahydrochloride to detect signal and hematoxylin as a counter-stain. Human first-trimester villi were used as positive control. The experiments were performed in five normal pregnancy tissues [25, 27–30].

### Isolation and culture of human EECs

Collected endometrial tissues were cut into small pieces after blood clot clearing and digested with 0.1% collagenase IV (Invitrogen, Carlsbad, CA, USA) for 1 h at 37 °C on a shaker. The supernatant was passed through a 38-μm sieve, and the reverse side of the sieve was then washed with DMEM, which was collected and centrifuged at 1000 g for 15 min. The resulting pellet was diluted with DMEM containing 15% heat-inactivated fetal bovine serum (FBS), 100 IU/ml penicillin and 100 μg /ml streptomycin and then seeded on a culture flask. After culture for 4 h, the suspension containing non-attached cells was collected and seeded on 6-well plates pre-coated with extracellular matrix (BD Matrigel™, Basement Membrane Matrix, USA). With this method, the purity of the cultured EECs can reach 90% according to our previous immunocytochemistry (ICC) identification (positive for CK7 and negative for vimentin) [31, 32].

### Immunofluorescence

The isolated EECs were seeded on 6-well plates which were pre-coated with coverslips at a density of 2 × 10 ^6^ cells/ml × 2 ml per well. At 80–90% confluence, the cells were fixed in 4% paraformaldehyde and treated with 0.2% Triton X-100. After blocking with goat serum for 2 h at room temperature, the cells were incubated with mouse anti-human CXCR4 (25 μg/ml, MAB172, R&D Systems, Abingdon, UK) and rabbit anti-human CXCL12 antibodies (20 μg/ml, ab168825, Abcam, Cambridge, MA, US). After three washes with PBS, the cells were incubated with secondary immunofluorescence antibody (Alexa Flour 488 goat anti-mouse, 1:200 and Alexa Flour 594 goat anti-rabbit, 1:200, SZGB-BIO, Beijing, China) at room temperature for 1 h in the dark and then incubated with 4′,6-diamidino-2-phenylindole (DAPI, Solarbio, S2110, Beijing, China) for 5 min (min) at 37 °C. Images were captured with an Olympus fluorescence microscope (Olympus, Tokyo, Japan). The experiments were performed using three independent samples [28].

### Preparation of EEC-conditioned medium

Freshly isolated EECs were seeded in 6-well plates pre-coated with extracellular matrix at a density of 2 × 10^6^ cells/ml per well and cultured continuously for 48 h. The supernatants, namely EEC-CM (conditioned medium), were collected and centrifuged at 2000 g and then stored at − 70 °C.

### Elisa

Freshly purified EECs were seeded in 24-well plates pre-coated with extracellular matrix at a density of 5 × 10^5^ cells/ml. The supernatants were collected at 24, 48 and 72 h of culture, respectively. A human CXCL12 enzyme-linked immunosorbent assay (ELISA) kit (R&D Systems, Abingdon, UK) was used to measure the chemokine levels in each supernatant sample according to the manufacturer’s instructions. The ELISA was performed in three separate samples [25, 26].

### Quantitative real-time PCR

Total RNA was extracted from primary EECs by using TRIZOL (Invitrogen, #10296010, USA). The cDNA was obtained by using the FastQuant RT Kit (with gDNAase) (TIANGEN, Beijing, China) according to the manufacturer’s instructions and then amplified with a fluorescence ratio polymerase chain reaction (PCR) instrument (Roche 96) in a reaction system (Beijing Yuan Quan Yi Ke Bio-Tech Co., Ltd., Beijing, China) with a final volume of 20 μl containing cDNA (1 μl), SYBR FAST qPCR Master Mix (10 μl), and specific primers (0.6 μl). A 5 min pre-cycle at 95 °C was followed by 40 cycles of 15 s at 95 °C, 20 s at 60 °C and 15 s at 72 °C. The primer pairs for cDNA amplification were 5′-GAA AGC CAT GTT GCC AGA GC-3′ (forward) and 5′-AGC TTC GGG TCA ATG CAC A − 3′ (reverse) for human CXCL12 (122 bp); 5′-CCG AGG CCC TAG CTT TCT TC-3′(forward) and 5′-GAG GAT CTT GAG GCT GGA CC-3′(reverse) for human CXCR4 (128 bp); 5′-GGG GAG CCA AAA GGG TCA TCT-3′ (forward) and 5′-GAG GGG CCA TCC ACA GTC TTC T^− 3^′ (reverse) for human glyceraldehyde-3-phosphate dehydrogenase (GAPDH) (235 bp). RNase-Free ddH_2_O was used as a negative control. The housekeeping gene GAPDH served as an internal control. The final results are expressed as a percentage of the positive control group. The experiments were performed on three samples by the Beijing Yuan Quan Yi Ke Bio-Tech Co., Ltd. [25, 28].

### Migration and invasion assay

Transwell plates were used to evaluate the migration and invasion of EECs [25, 26, 32]. The isolated EECs were seeded on the upper chamber directly in the migration assay or on cell inserts (8 mm pore size, 6.5 mm diameter; Corning, NY, USA) pre-coated with 20 μl extracellular matrix in the invasion assay.

Firstly, freshly isolated EECs (2 × 10^5^ in 200 μl DMEM) were loaded into each transwell inserts and treated with rhCXCL12 (100 ng/ml, 300-28A-100, PeproTech, USA) or EEC-CM. Before this treatment, the cells in the wells were pre-incubated with CXCR4 neutralizing antibody (20 μg/ml, MAB172, R&D Systems, Abingdon, UK), CXCL12 neutralizing antibody (25 μg/ml, MAB310,R&D Systems, Abingdon, UK), PI3K-AKT inhibitor LY294002 (25 μM, #1130, Tocris Bioscience, UK) and ERK inhibitor U0126 (20 μM, #1144, Tocris Bioscience, UK), respectively. The lower chamber was filled with 800 μl DMEM containing 15% FBS. The cells were then incubated at 37 °C for 48 h. Cultures of EECs with vehicle were used as control.

Secondly, the inserts were removed and washed in PBS, and the non-invading cells were removed from the upper surface of the filter by cotton swab wiping. The inserts were fixed in 4% formalin, stained with hematoxylin and observed using an inverted phase contrast microscope (Olympus, Tokyo, Japan).

Migrated cells were counted at a magnification of 200. To eliminate individual variability, the results were assessed by two independent researchers, and the migratory/invasive index was calculated as the proportion of migrated cells in the experimental group relative to its own control. Samples were run in duplicate and experiments were performed using three different samples.

### In-cell Western blot

We used in-cell western blot to measure the levels of phospho-AKT (protein kinase B), phospho-ERK (extracellular regulated protein kinases) and phospho-PI3K (phosphatidylinositol 3-kinase). Freshly isolated EECs (2 × 10^4^/well) were seeded on 96-well plates. At 80–90% confluence, the cells were serum-starved in DMEM supplemented with 1% FBS for 12 h and treated with vehicle, CXCL12 (100 ng/ml) with or without CXCR4 (20 μg/ml), U0126 or LY294002 for 0, 1, 5, 10, 30, and 60 min. After stimulation, the cells were immediately fixed with 4% formaldehyde for 20 min at room temperature. After washing with 0.2% Triton, the cells were blocked with 150 μl of LI-COR Odyssey Blocking Buffer (LI-COR Biosciences, Lincoln, Nebraska, USA) for 90 min at room temperature. The primary antibodies used were rabbit anti-human phospho-ERK (Thr202/Thr204, Cell Signaling, Danvers, MA, #4370, 1:200), mouse anti-human total ERK (Cell Signaling, Cell Signaling, Danvers, MA, #9102, 1:50), rabbit anti-human phospho-AKT (Ser 473, Cell Signaling, Danvers, MA, #4060, diluted at 1:200), mouse anti-human total AKT (Cell Signaling, Danvers, MA, #4691, 1:50), rabbit anti-human phospho-PI3K (p85/p55, Abbkine, USA, Abp55592, 1:200) and mouse anti-human total PI3K (Proteintech, Wuhan, China, 60,225–1-1, 1:50). After treatment overnight at 4 °C, the wells were then incubated with the corresponding secondary IRDyeTM700DX-conjugated affinity purified anti-mouse fluorescent antibody (red fluorescence) or IRDyeTM800DX-conjugated affinity purified anti-rabbit fluorescent antibody (green fluorescence) recommended by the manufacturer (Rockland, Inc., Gilbertsville, PA, USA). This procedure was performed in the dark to avoid exposure to light. Images of the target protein were obtained using the Odyssey Infrared Imaging System (LI-COR, Inc. Lincoln, Nebraska, USA). The protein expression level was calculated as the ratio of the fluorescence intensity of the target protein to that of the corresponding control. The experiments were performed in duplicate in three samples [32, 33].

### Statistics

Statistical comparisons of data from various groups were preformed using the one-way ANOVA analysis followed by post hoc analysis with the least significant difference (LSD) test when variances of the subgroups of data are equal or Dunnett’s T3 when variances of the subgroups have significantly different. The results are expressed as the means ± SD. Inter-group differences were considered statistically significant at *P* < 0.05.

## Results

### Expression of CXCL12/CXCR4 in human first-trimester EECs

**Fig. 1:**
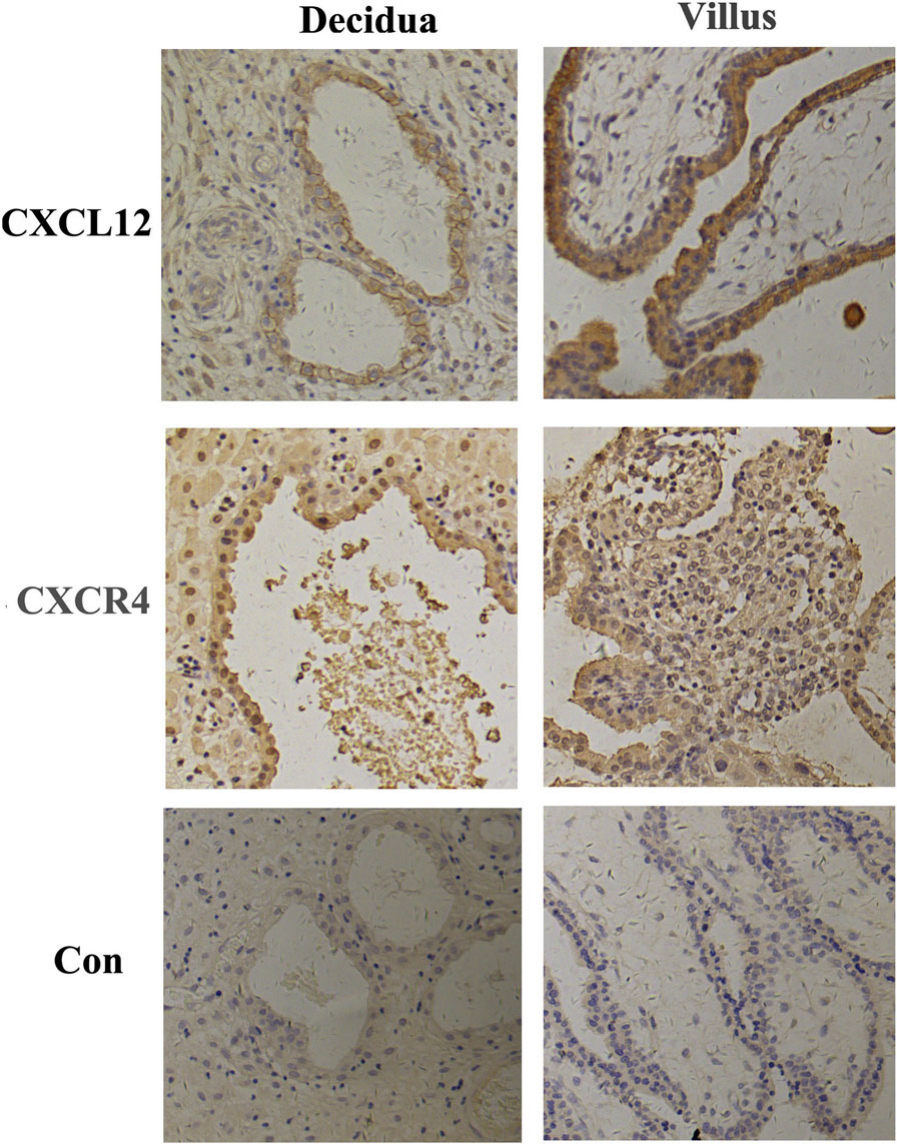
CXCL12 and CXCR4 were both expressed in human first-trimester EECs in vivo. Expression of CXCL12 and CXCR4 proteins in human first-trimester endometrial epithelial cells (EECs) of decidual tissues was measured with immunohistochemical analysis. CXCL12 and CXCR4-specific brown-colored staining was clearly observed in the cytoplasma of EECs (*n* = 5). No specific staining was observed in the negative control. Human first-trimester villi were used as positive controls

**Fig. 2:**
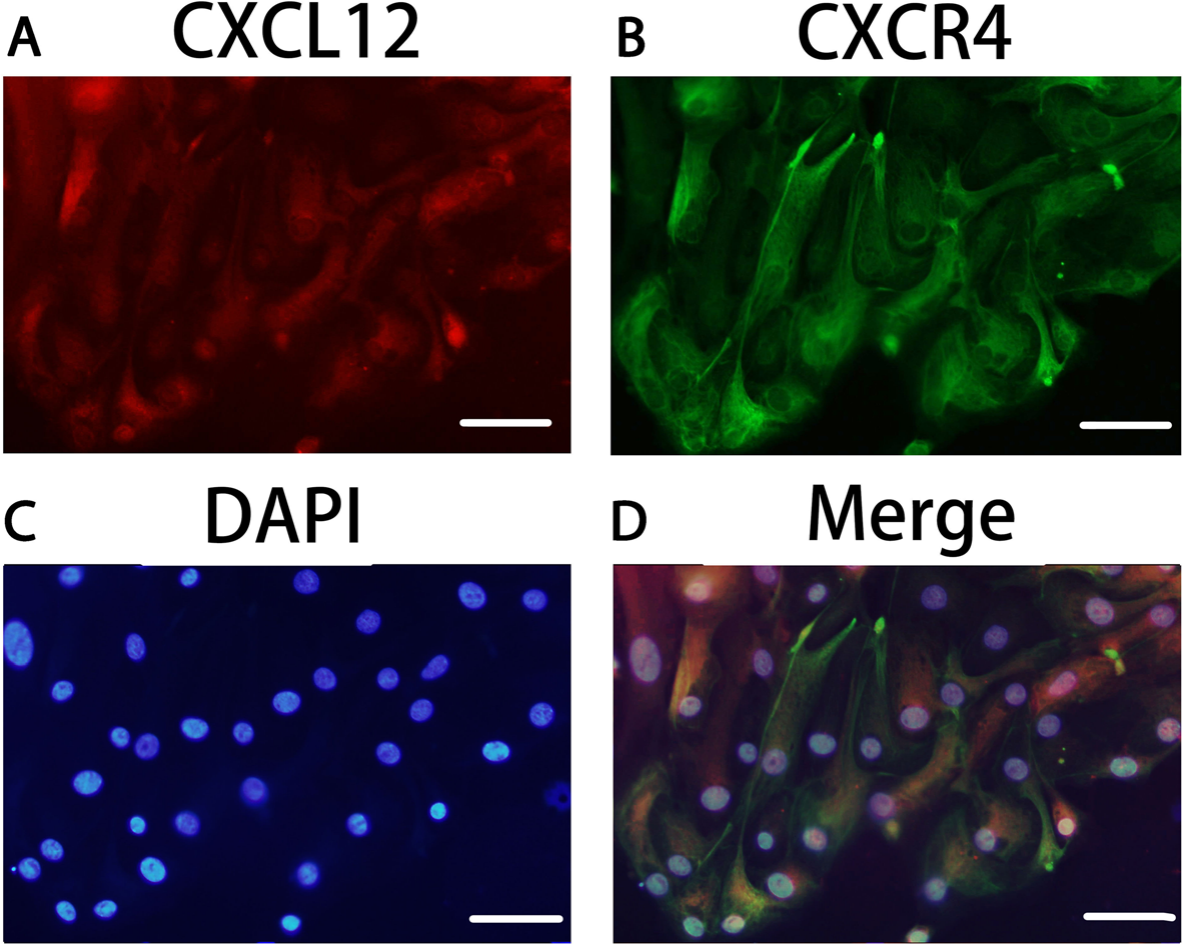
CXCL12 and CXCR4 were both expressed in primary human first-trimester EEC cultures in vitro. Immunofluorescence staining of both CXCL12 and CXCR4 protein was detected in the primary cultured human first-trimester endometrial epithelial cells (EECs). **a**: CXCL12 expression (red fluorescence) in EECs. **b**: CXCR4 expression (green fluorescence) in EECs. **c**: DAPI staining in EECs. **d**: Merged picture. Magnification: × 400

**Fig. 3:**
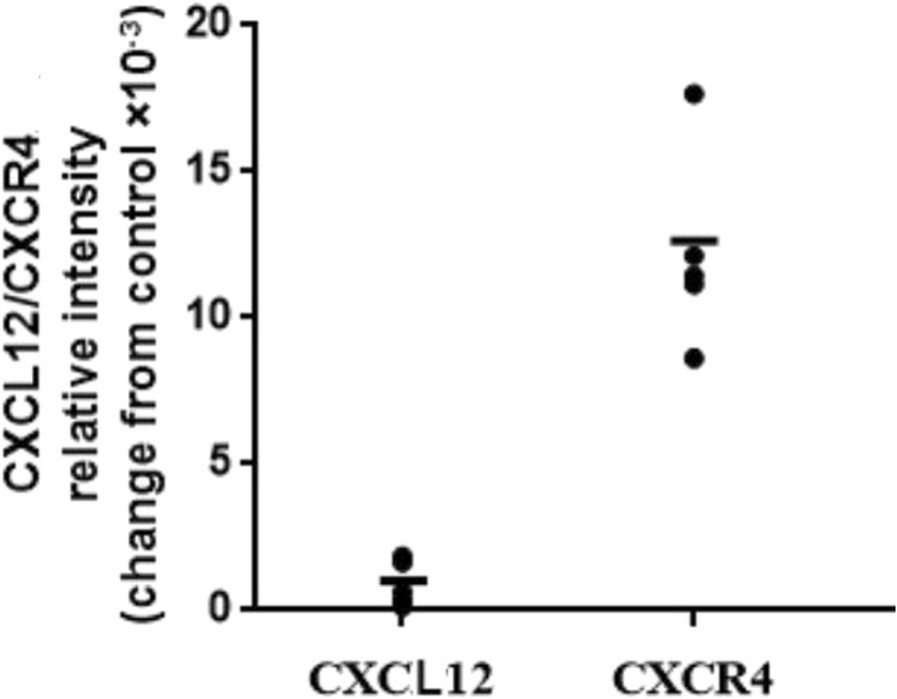
Expression of CXCL12 and CXCR4 mRNAs in primary human first-trimester EECs. The expression of CXCL12 and CXCR4 mRNA in human first-trimester endometrial epithelial cells (EECs) was measured by real-time polymerase chain reaction (PCR). The levels of CXCL12 and CXCR4 mRNA were equal to the ratio of the absorbance of the target gene to that of GAPDH. The results showed the relative levels of CXCR4 and CXCL12 mRNA in 6 samples and the horizontal line represents the average mRNA level of CXCL12 and CXCR4 in EECs

CXCL12 and CXCR4-specific brown-colored staining was seen in the cytoplasm of human primary first-trimester EECs in IHC staining, as shown in Fig. 1. The immunofluorescence in Fig. 2 showed positive CXCL12 and CXCR4 protein expression in human EECs cultured in vitro. Red fluorescence (CXCL12) and green fluorescence (CXCR4) were both detected in these cells. The real-time PCR also confirmed positive CXCL12 and CXCR4 transcription in human first-trimester EECs as shown in Fig. 3, with mean mRNA levels of 0.0012 and 0.0142, respectively, changed from positive control.

**Fig. 4:**
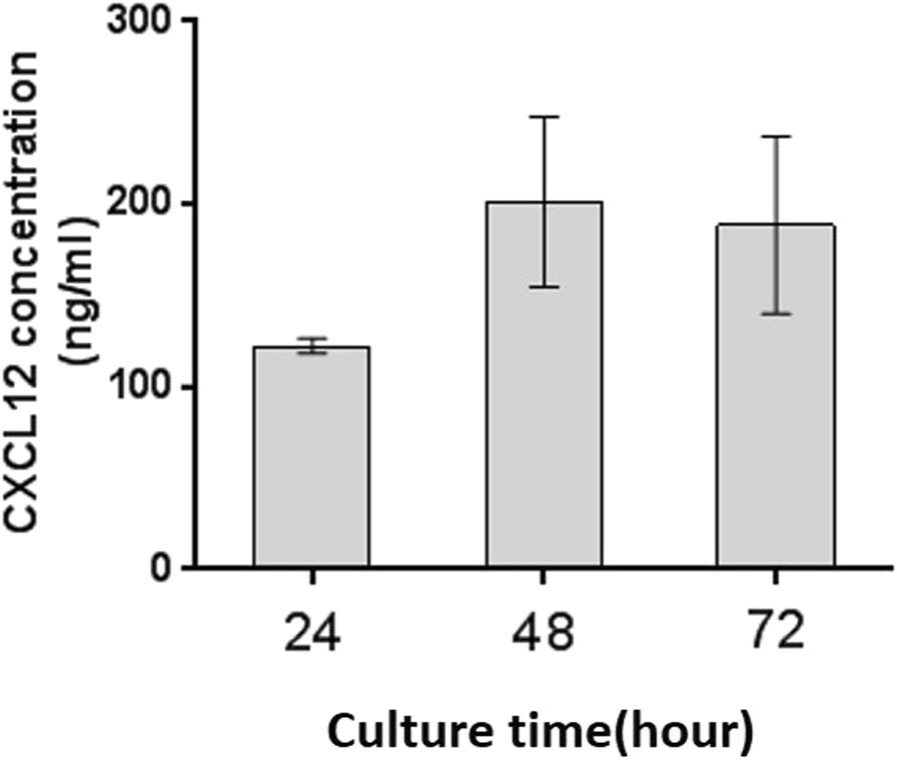
Primary human first-trimester EEC cultures spontaneously secreted CXCL12 in vitro. Spontaneous CXCL12 secretion in the supernatants of in vitro cultured human first-trimester endometrial epithelial cells (EECs) was measured at 24, 48 and 72 h with enzyme-linked immunosorbent assay (ELISA). It was shown that human first-trimester primary cultured EECs continuously secreted CXCL12 in vitro. Data are presented as the mean ± SD (*n* = 3)

The soluble CXCL12 was found in the supernatant of EECs as shown in ELISA assays (Fig. 4) that showed continuous secretion of CXCL2 in the primary culture of human first-trimester EECs in vitro. The cumulative concentration of CXCL12 was 122.24 ± 4.06 pg/ml, 201.06 ± 46.44 pg/ml and 188.47 ± 48.65 pg/ml at 24, 48 and 72 h of culture, respectively (Fig. 4).

### CXCL12 promoted the migration and invasion of human first-trimester EECs via CXCR4 in an autocrine manner

**Fig. 5:**
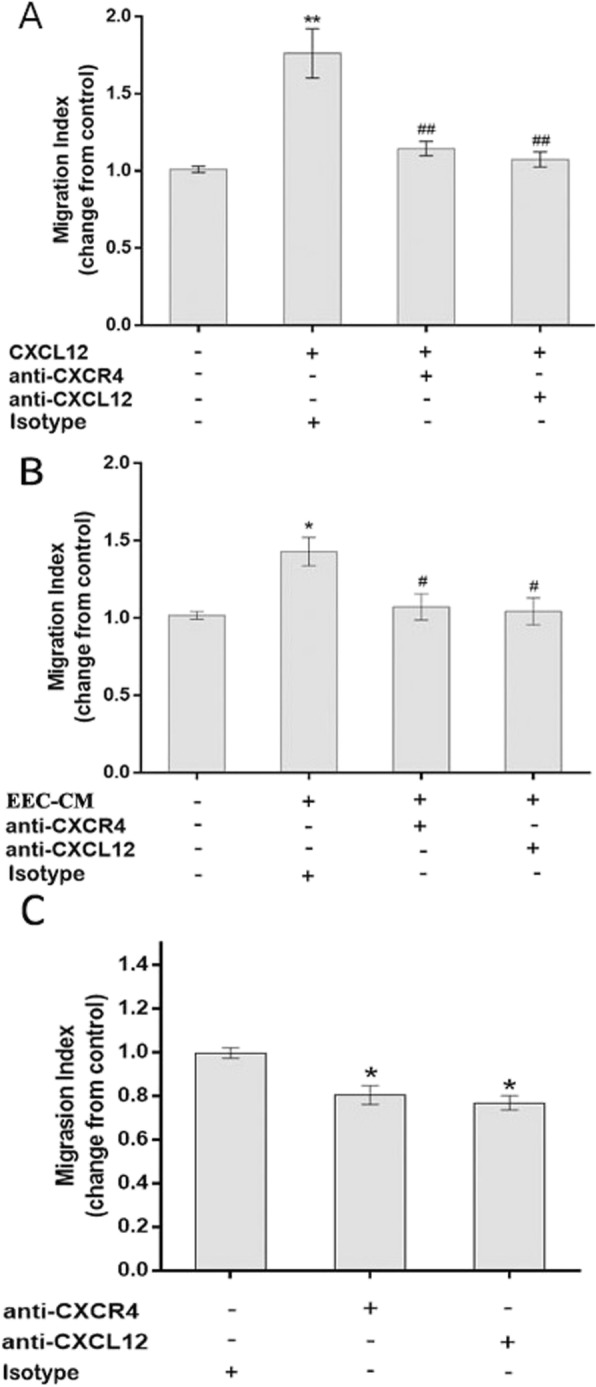
EEC-derived CXCL12 induced EEC migration by binding to CXCR4 **a** Exogenous CXCL12 significantly increased EEC migration in vitro*,* whereas neutralizing antibodies against CXCR4 or CXCL12 effectively inhibited the CXCL12-induced migration of EECs. **b** EEC-CM significantly increased EEC migration in vitro*,* and a neutralizing antibody against CXCR4 or CXCL12 effectively inhibited the EEC-CM-induced migration of EECs. **c** CXCR4 or CXCL12 blocking antibody alone could markedly inhibit the migration of EECs. * *P* < 0.05, ** *P* < 0.01 vs. control; # P < 0.05, ## P < 0.01, vs. CXCL12-treated or EEC-CM treated group. Data are presented as mean ± SD (n = 3). EEC: human first-trimester endometrial epithelial cell, EEC-CM: EEC conditioned culture medium

In the migration assay, similar to exogenous CXCL12 (1.83 ± 0.13 vs 1.00 ± 0.04, *P* < 0.05), EEC-CM (1.43 ± 0.09 vs 1.00 ± 0.09, P < 0.05) significantly increased the migration of human first-trimester EECs in vitro compared to the corresponding control, as shown in Fig. 5. The migration induced by CXCL12 (1.83 ± 0.13 vs 1.06 ± 0.04 and 1.10 ± 0.03, P < 0.05) or EEC-CM (1.43 ± 0.09 vs 1.04 ± 0.09 and 1.07 ± 0.08, *P* < 0.01) was clearly inhibited when the neutralizing antibodies against CXCL12 or CXCR4 was added.

**Fig. 6:**
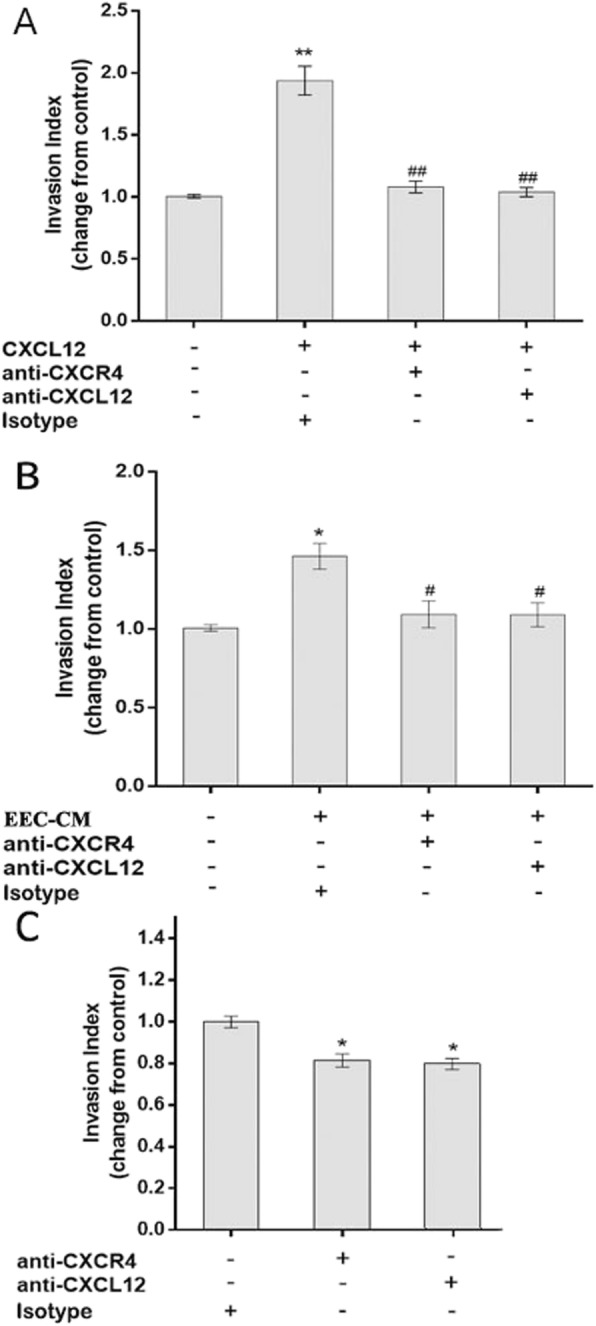
EEC-derived CXCL12 increased EEC invasion by binding to CXCR4 **a** Exogenous CXCL12 significantly increased EEC invasion in vitro*,* whereas neutralizing antibody against CXCR4 or CXCL12 effectively inhibited the CXCL12-stimulated invasion of EECs. **b** EEC-CM significantly increased EEC invasion in vitro*,* and a neutralizing antibody against CXCR4 or CXCL12 effectively inhibited EEC-CM-induced invasion of EECs. **c** CXCR4 or CXCL12 blocking antibody alone markedly inhibited the invasion of EECs. * P < 0.05, ** P < 0.01, compared to the control; # P < 0.05, ## P < 0.01, compared to the CXCL12-treated or EEC-CM-treated group. Data are presented as mean ± SD. (n = 3). EEC: human first-trimester endometrial epithelial cell, EEC-CM: EEC conditioned culture medium

The invasion assay confirmed the findings in the migration assay. As shown in Fig. 6, the invasive ability of EECs was significantly increased by exogenous CXCL12 (1.94 ± 0.12 vs 1.00 ± 0.05, P < 0.01) and EEC-CM (1.46 ± 0.08 vs 1.00 ± 0.09, P < 0.01) compared to the corresponding control. Neutralizing antibodies against CXCR4 or CXCL12 effectively blocked the CXCL2-induced (1.94 ± 0.12 vs 1.08 ± 0.05 and 1.04 ± 0.04, P < 0.01) and EEC-CM-induced EEC invasion (1.46 ± 0.08 vs 1.09 ± 0.09 and 1.09 ± 0.08, P < 0.01).

To further explore the mechanism of CXCL12 in inducing the migration and invasion of EECs, we also evaluated the migratory and invasive ability of EECs in the presence of CXCR4 or CXCL12-neutralizing antibody alone (Fig. 5c, Fig. 6c) and found that the migration and invasion of EECs significantly decreased with the presence of CXCL12 (1.00 ± 0.05 and 1.00 ± 0.03 vs 0.77 ± 0.03 and 0.82 ± 0.03 P < 0.01 or CXCR4 (0.80 ± 0.02 and 0.80 ± 0.03, P < 0.01) neutralizing antibody.

### CXCL12 activated the PI3K, AKT and ERK1/2 pathways in EECs by binding to CXCR4

**Fig. 7:**
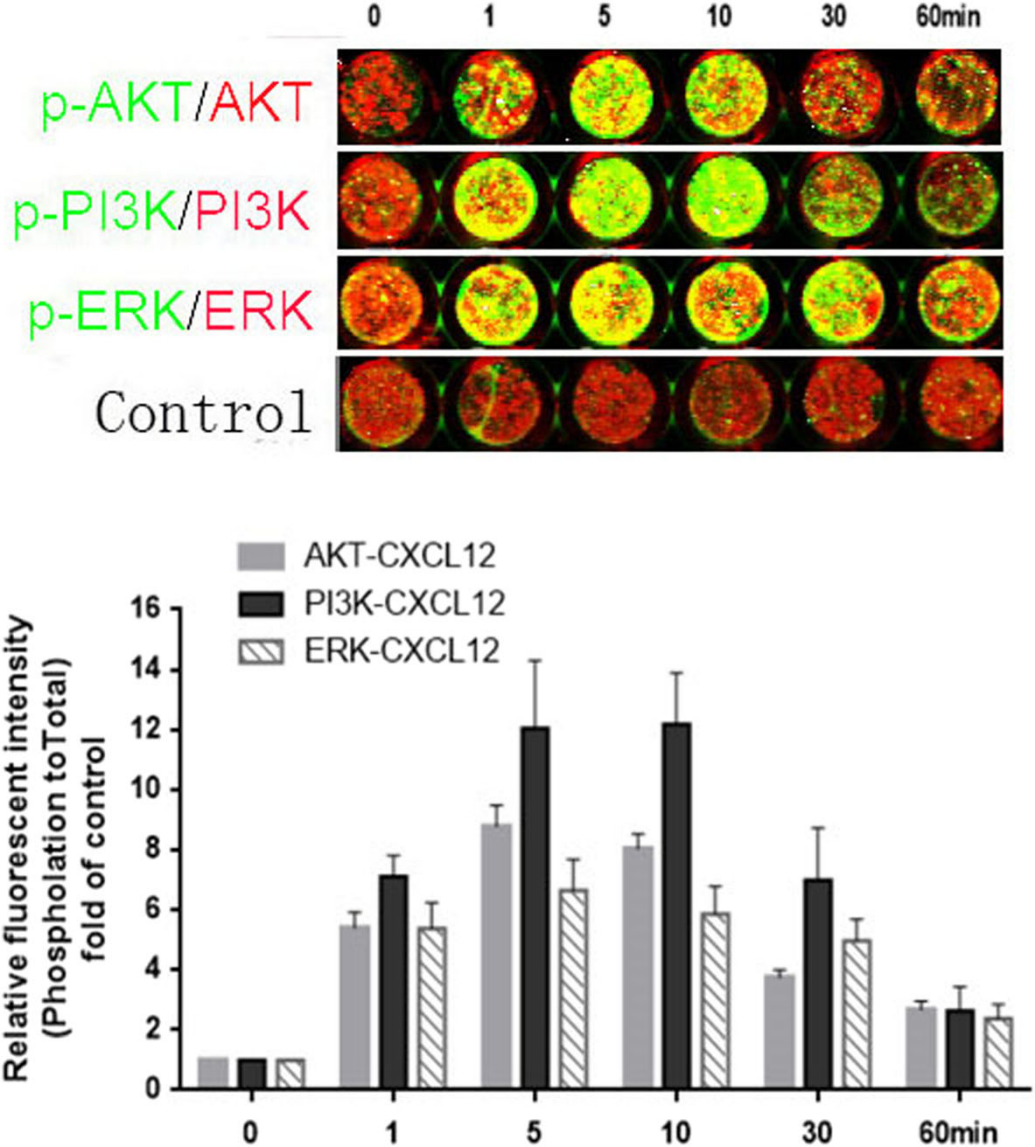
CXCL12 stimulated the activation of Akt, PI3K and ERK in EECs. Human first-trimester endometrial epithelial cells (EECs) were serum starved for 12 h and then stimulated with CXCL12 (100 ng/ml). The phosphorylation of protein kinase B (AKT), extracellular regulated protein kinases (ERK) and phosphatidylinositol 3-kinase (PI3K) were evaluated with in-cell western blot analysis. CXCL12 (at 100 ng/ml) led to time-dependent increase in phosphorylation of AKT, PI3K and ERK1/2 in EECs. Phosphorylated proteins were stained in green and total proteins stained in red. The phosphorylated to total protein ratio was normalized to 1 in the untreated control (n = 3)

To further identify the downstream signals of the CXCL12/CXCR4 axis, an in-cell western blot was used to determine the phosphorylation level of AKT, PI3K and ERK1/2 in EECs after stimulation with CXCL12 for 1, 5, 10, 30, or 60 min. CXCL12 at 100 ng/ml induced phosphorylation of AKT, PI3K and ERK1/2 in first-trimester human EECs in various manners (Fig. 7).

The phosphorylation of ERK1/2 in EECs treated with CXCL12 increased significantly at 1 min till the peak level at 5 min and then decreased at 60 min. The significantly increased phosphorylation of PI3K and Akt at 1 min was sustained for at least 10 min before the decrease at 30 min (Fig. 7).

**Fig. 8:**
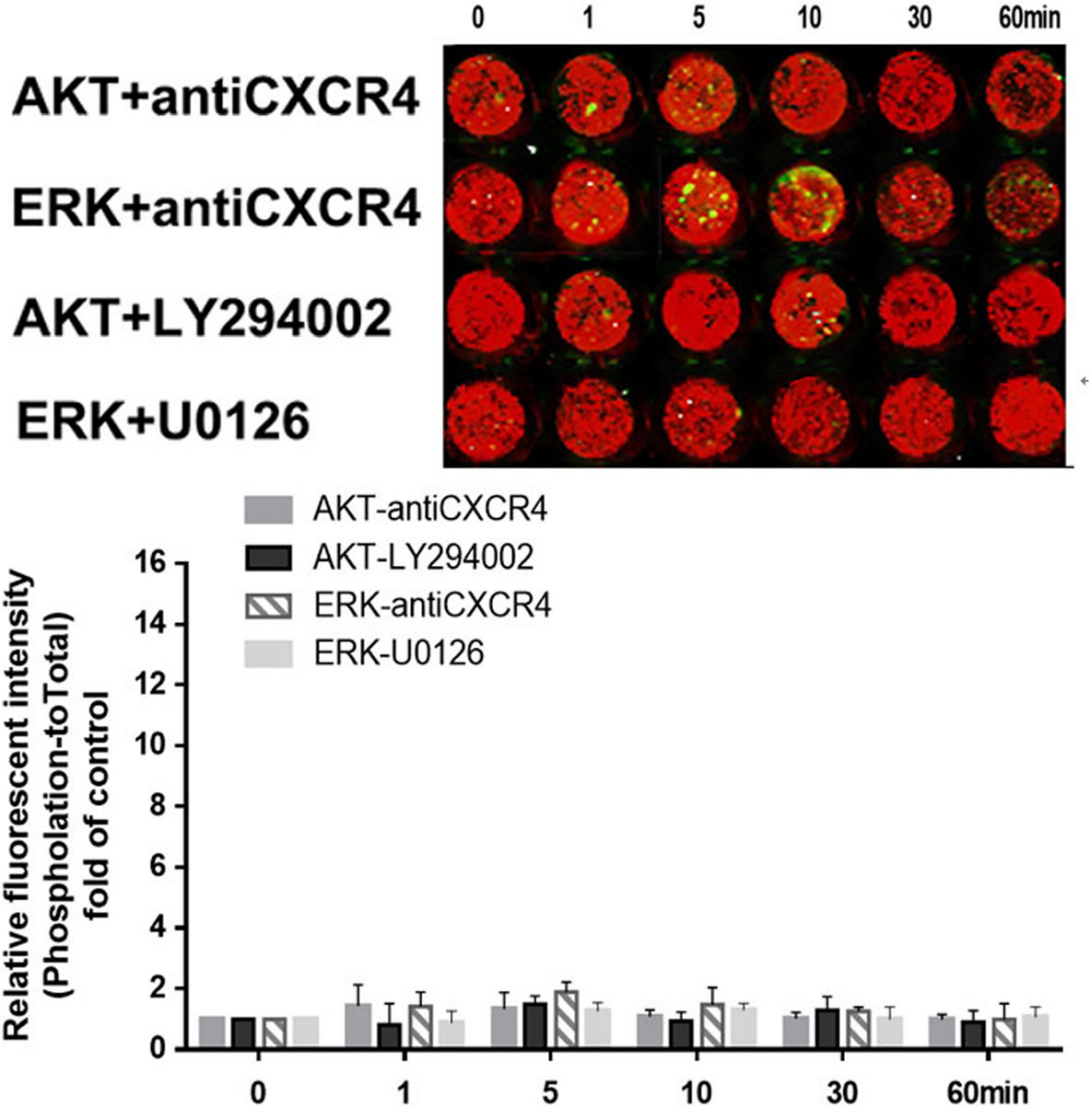
CXCL12 stimulated the activation of Akt, PI3K, and ERK in EECs by binding to CXCR4. The phosphorylation of protein kinase B (AKT) and extracellular regulated protein kinases (ERK) in human first-trimester endometrial epithelial cells (EECs) was inhibited by treatment with CXCR4 neutralizing antibody, LY294002 (a PI3K/AKT blocker) or U0126 (a ERK1/2 blocker). Phosphorylated proteins were stained in green and total proteins were stained in red. The phosphorylated to total protein ratio was normalized to 1 in the untreated control (n = 3)

As shown in Fig. 8, LY294002 that inhibits PI3K/AKT inhibitor, U0126 that inhibits MEK, and CXCR4 neutralizing antibody all had similarly effective blocking effect on the CXCL12-induced phosphorylation of AKT and ERK1/2.

### CXCL12/CXCR4 promoted EEC migration and invasion by activating the AKT/PI3K signaling pathway

**Fig. 9:**
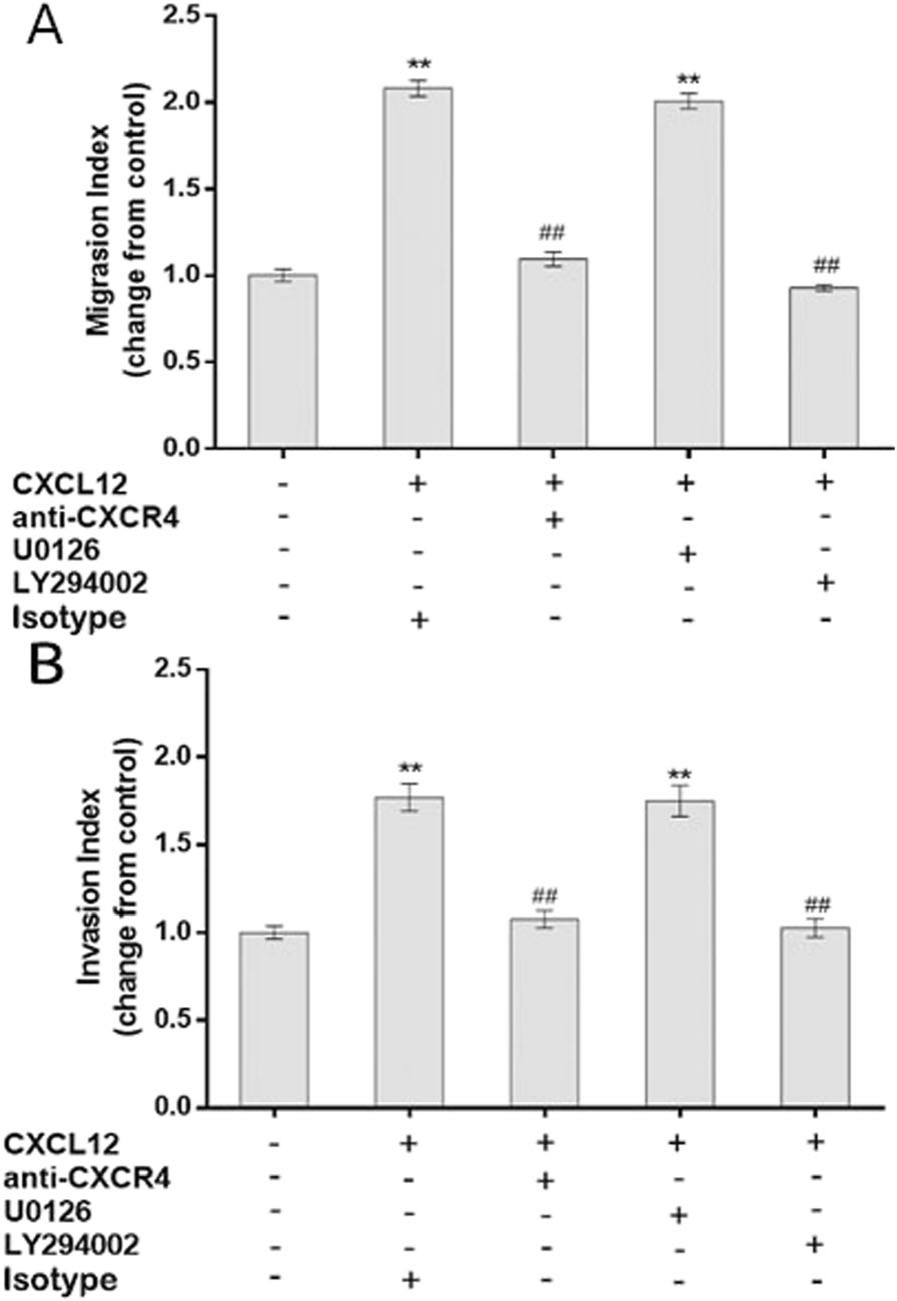
CXCL12 stimulated the migration and invasion of EECs by activating AKT/ PI3K signaling. Exogenous CXCL12 significantly increased the migration and invasion of EECs in vitro*,* which effect was remarkably inhibited by neutralizing antibodies to CXCR4 or PI3K/AKT blocker (LY294002). The ERK1/2 blocker (U0126) failed to block the CXCL12-induced EEC migration or invasion. * P < 0.05, ** P < 0.01 vs. control; # P < 0.05, ## P < 0.01 vs. CXCL12-treated or EEC-CM-treated group. Data are presented as mean ± SD. (n = 3). EEC: human first-trimester endometrial epithelial cell. EEC-CM: EEC conditioned medium

To study the role of PI3K, AKT or ERK1/2 signaling in regulating the CXCL12-mediated migration and invasion of EECs, we incubated EECs with rhCXCL12, CXCR4 neutralizing antibody, LY294002 and U0126, separately. As shown in Fig. 9, both CXCR4 neutralizing antibody and PI3K/AKT blocker but not U0126 significantly reduced CXCL12-mediated EEC migration and invasion (CXCR4 neutralizing antibody or PI3K/AKT blocker vs. CXCL12, *P* < 0.05 for both CXCR4 neutralizing antibody and PI3K/AKT blocker; U0126 vs. CXCL12, *P* > 0.05).

## Discussion

Many studies are concerned with the role of EECs in blastocyst implantation while it is easy to overlook other functions of EECs at the maternal-fetal interface. In the present study, we found that except for CXCR4, CXCL12 is also expressed in human first-trimester EECs in situ and in vitro at both the mRNA and protein levels. According to reported studies, CXCL12 and CXCR4 may be crucial chemokines and chemokine receptors involved in implantation and early pregnancy [10–18, 25–28, 34, 35]. EECs are an intrinsic component of the functional endometrium. To examine the role of EECs at the maternal-fetal interface during pregnancy via the CXCL12/CXCR4 signaling pathway, we used exogenous CXCL12 treatment as control and measured the effect of EEC-CM on the migration and invasion of EECs in vitro. Similar to exogenous CXCL12, EEC-CM significantly induced the migration and invasion of EECs, which was effectively blocked by neutralizing antibodies against CXCR4 or CXCL12 and the presence of CXCL12 or CXCR4 neutralizing antibody alone effectively inhibited the invasion and migration of EEC under natural condition. This result is consistent with our hypothesis that EECs can self-adjust their own motility via the CXCL12/CXCR4 axis. As pregnancy progresses, the endometrial glands will gradually atrophy. Thus, except for involvement in embryo implantation, other functions of EEC have not been investigated. However, our result that EEC possesses the ability to modulate their own motility suggests the function of these cells is not fully understood and the complexity of the materal-fetal interface still deserves further study.

Researchers often consider the relationship between the fetus and mother from the TC-oriented perspective and pay close attention to the modulation of endometrial function by fetus-derived signals. However, this study clearly demonstrated that human first-trimester EECs can also modulate their own motility without the stimulation of TC in an autocrine manner by expressing some specific ligands and receptors during early pregnancy. Several studies have confirmed the replacement and reconstruction of uterine glands by endoglandular TCs, which is similar to the remodeling of spiral arteries prior to the onset of maternal circulation at the end of the first trimester [36, 37]. An in vitro co-culture model has confirmed that DSCs, another main maternal derived cells at the maternal fetal interface, can become more motile and migrate away from the implantation site, allowing the embryo to spread out [38]. Our previous study also found that TC-derived CXCL12 was able to reinforce the motile ability of DSCs via CXCR4 ligation during first-trimester pregnancy [26]. Therefore, it is reasonable to infer that just like the DCSs, an autonomous motility of EECs may represent an efficient method of communication and cooperation with TCs, through which the endomtrium can better coordinate with embryo. Whether EECs motility is related to abnormal pregnancy needs further researches.

In fact, our previous studies unveiled the role of the TC-derived CXCL12, not only in TC invasion in an autocrine manner but also in DSC motility in a paracrine manner via CXCR4 ligand [25, 26]. Other groups also indicated the role of CXCL12/CXCR4 in the establishment of a unique immune microenviroment during early pregnancy [10–18, 34, 35]. This study further confirmed the modulation of CXCL12/CXCR4 axis in an autocrine manner in the context of EEC migration and invasion. Therefore, we suggested that CXCL12/CXCR4-mediated signaling might be a bridge for communication in different cells via different patterns, which contributes to improving the crosstalk and synchronization of different types of cells at the maternal-fetal interface. Unlike DSCs that mainly express CXCR4 [25, 26], EECs produced both CXCL12 and CXCR4 as indicated in the present study. Whether the difference in expression patterns of CXCL12 and CXCR4 in different cells could translate into cell-specific different functions is still unknown and deserves further investigation.

To better understand the potential molecular mechanisms of CXCL12/CXCR4 ligation, we measured the phosphorylation levels of AKT, ERK and PI3K in EECs treated with CXCL12. In this study, CXCL12 activated PI3K/AK signaling and the increased phosphorylation level of PI3K/AKT could be effectively inhibited by CXCR4 neutralizing antibody and PI3K-AKT blocker LY294002. LY294002 showed inhibitory effect on CXCL12 or EEC-CM-induced EEC migration and invasion, which is very similar to the role of CXCR4 in EECs. Thus, it is suggested that CXCL12 promotes EEC migration and invasion via CXCR4 ligation through the activation of PI3K-AKT signaling in an autocrine manner. Although CXCL12 activated the ERK in a time-dependent manner, U0126, an ERK inhibitor, failed to block the increased motility induced by CXCL12 or EEC-CM. Therefore, biological activities might be mediated by different signaling molecules, which further suggests the complexity of biological activity at the maternal-fetal interface. The significance of ERK signal activation in EECs requires further investigation.

The limitation of the study was that the samples were collected from unintended pregnancy and it was impossible to track the pregnancy outcomes of these volunteers. Only three to five samples were used in each assay because of the rarity of clinical samples. To better understand the role of CXCL12/CXCR4 and EEC motility in pregnancy, in the future research, we may explore their relationship with implatation failure and miscarriage, which is beneficial to find a biological target for unexplained miscarriage.

## Conclusion

In conclusion, evidence obtained in this study supported that human first-trimester EECs promote their own motility in an autocrine manner via the CXCL12/CXCR4 axis through the activation of PI3K/AKT signaling, suggesting the ability of EECs to self-modulate their biological activity during early pregnancy. These discoveries contribute to a better understanding of the pregnancy physiology and provide new insight on the etiology of pregnancy complications including implantation failure and unexplained miscarriage.

## Data Availability

The datasets used and/or analyzed during the current study are available from the corresponding author on reasonable request.

## Abbreviations

AKT: Protein kinase B
CXCL12: C-X-C motif chemokine ligand 12
CXCR4: C-X-C chemokine receptor type 4
DAB: 3, 30-diaminobenzidine tetrahydrochloride
DMEM: Dulbecco’s Modified Eagle’s Medium
DSC: Decidual stroma cells
EEC: Endometrial epithelial cell
ELISA: Enzyme-linked immunosorbent assay
ERK: Extracellular regulated protein kinases
FBS: Fetal bovine serum
ICC: Immunocytochemistry
IHC: Immunohistochemistry
PBS: Phosphate-buffered saline
PCR: Polymerase chain reaction
PI3K: Phosphatidylinositol 3-kinase
RhCXCL12: Recombinant human CXCL12
TBST: Tris-buffered saline with Tween 20
TC: Trophoblast cell
TC-CM: TC-conditioned medium
